# *MPDZ* Pathogenic Variants Cause Obstructive Ventriculomegaly Related to Diencephalosynapsis and Third Ventricle Atresia

**DOI:** 10.3390/genes16060707

**Published:** 2025-06-13

**Authors:** Sara Cabet, Jean-François Ghersi-Egea, Suonavy Khung-Savatovsky, Fabien Guimiot, Audrey Putoux, Isabelle Sabatier, Carla Fernandez, Laure Raymond, Jérémie Mortreux, Hélène Laurichesse Delmas, Fabrice Eric Cuillier, Fabien Ho, Gaetan Lesca, Jean-Luc Alessandri, Laurent Guibaud

**Affiliations:** 1 Pediatric and Fetal Imaging Department, Femme-Mère-Enfant Hospital, Hospices Civils de Lyon, Claude Bernard Lyon 1 University, 69500 Lyon, France; 2 Institut NeuroMyoGène, CNRS UMR5292, INSERM U1028, Claude Bernard Lyon 1 University, 69500 Lyon, France; gaetan.lesca@chu-lyon.fr; 3 Fluid Team, Lyon Neurosciences Research Center, CNRS UMR5292, INSERM U1028, Claude Bernard Lyon 1 University, 69500 Lyon, France; 4 Functional Unit of Fetal Pathology, Department of Genetics, Robert-Debré Hospital, AP-HP, 75019 Paris, Francefabien.guimiot@aphp.fr (F.G.); 5 INSERM U1141, Paris-Cite University, 75019 Paris, France; 6 Department of Genetics, Groupement Hospitalier Est, Hospices Civils de Lyon, Claude Bernard Lyon 1 University, 69500 Lyon, France; 7 Department of Pediatric Neurology, Femme-Mère-Enfant Hospital, Hospices Civils de Lyon, 69500 Lyon, France; 8 Department of Pathology, Felix Guyon Hospital, 97490 Saint-Denis, France; 9 Genetic Department, Eurofins Biomnis, 69500 Lyon, France; 10 Multidisciplinary Prenatal Diagnosis Center, University Hospital Center of Clermont Ferrand, 63000 Clermont Ferrand, France; 11 Fetal Imaging Department, Felix Guyon Hospital, 97490 Saint-Denis, France; 12 Imagerie Capricorne, Clinique Sainte-Clotilde, 97490 Saint-Denis, France; 13 Genetic Department, Felix Guyon Hospital, La Réunion University, 97490 Saint-Denis, France

**Keywords:** diencephalosynapsis, hydrocephalus, *MPDZ*, thalamus, third ventricle, ventriculomegaly

## Abstract

Objective: Ventriculomegaly is the main prenatal imaging feature for diagnosing fetal central nervous system anomalies in humans. Many ventriculomegalies can be related to genetic causes, regardless of their imaging presentations. Among these, *MPDZ* variants have been reported to cause severe ventriculomegaly inherited in an autosomal recessive manner (OMIM#615219). Several hypotheses have been put forward linking *MPDZ* variants to ventriculomegaly, but the precise underlying mechanisms, in particular whether its origin is obstructive or non-obstructive, are yet to be elucidated. Methods: To address this question, we retrospectively analyzed pre- and postnatal neuro-imaging and neuropathological data for cases of ventriculomegaly in which *MPDZ* variants were found through exome or genome sequencing. We performed anti-MPDZ immunostaining on fetal brain samples. Results: We analyzed six cases (four fetuses and two children) of ventriculomegaly of variable severities with *MPDZ* variants. The precise analysis of brain MRI data, corroborated by fetopathological examinations, demonstrated an obstructive pattern of ventriculomegaly upstream from partial fusion of the thalami, also called diencephalosynapsis, with partial atresia of the third ventricle, which could extend to Sylvius’s aqueduct. Conclusions: The morphological analysis using targeted brain magnetic resonance imaging (MRI) and neuropathological data allowed us to unravel the underlying mechanisms of congenital ventriculomegaly related to *MDPZ* variants.

## 1. Introduction

Ventriculomegaly is the main prenatal imaging feature for diagnosing fetal central nervous system (CNS) anomalies. Once ventriculomegaly is confirmed and measured using precise standardized guidelines, the prognosis of this feature is not related to its minor, moderate, or severe size but mainly to its underlying etiology. Minor ventriculomegaly represents, in the vast majority, a variant of normal, often called “isolated”, ventriculomegaly [1]. This latter diagnosis is, by definition, a provisional diagnosis of exclusion after ruling out underlying CNS malformations of clastic or tumoral causes, as well as syndromic entities, through a systematic analysis of prenatal brain imaging. This analysis focuses both on anatomy by looking closely at the ventricular system, gyration, and peri-cerebral spaces and on the identification of specific imaging patterns suggestive of obstructive, infectious, or vascular clastic origins [2,3]. In addition, many ventriculomegalies can be related to genetic causes, regardless of their imaging presentations. Among these, *MPDZ* variants have been reported to cause severe ventriculomegaly inherited in an autosomal recessive manner (OMIM#615219). This gene encodes the MPDZ protein, or the multiple PDZ (PSD95, DLG, ZO-1) domain protein, which is a multidomain protein located in the cellular tight junctions of the epithelial cells, including those forming the choroid plexuses. Several hypotheses have been put forward linking *MPDZ* variants to ventriculomegaly, but its precise underlying mechanisms, in particular whether its origin is obstructive or non-obstructive, are yet to be elucidated.

To address this question, we analyzed six cases on the basis of pre- and postnatal neuro-imaging in which *MPDZ* pathogenic variants were found. The six cases, which presented with ventriculomegaly of variable severities, encompassed four fetuses and two children from three families. The morphological analysis using targeted brain magnetic resonance imaging (MRI) and neuropathological data allowed us to unravel the underlying mechanisms of congenital ventriculomegaly related to *MDPZ* variants. Anti-MPDZ immunostaining was performed in one case. We also provide data on the postnatal outcomes, which are of great importance both to the identification of imaging patterns suggestive of such variants and to prenatal counselling when the etiology of ventriculomegaly has been identified.

## 2. Materials and Methods

Multicentric collaboration allowed us to collect data for three families with fetuses and children carrying genetic variants of the *MPDZ* gene. Appropriate consent was obtained from our local ethics committee (CSE-HCL; approval code: 25-5211). Family 1 benefited from genome sequencing in quatuor on the French sequencing platform Auragen (Lyon, France) using a NovaSeq 6000 (Illumina, San Diego, CA, USA), and Family 2 and Family 3 benefited from exome sequencing (respectively, Eurofins Biomnis, Lyon, and CHU, Clermont-Ferrand) using NextSeq (Illumina, San Diego, CA, USA), after the use of a normal SNP array on amniotic fluid.

### 2.1. Genome Sequencing

Family 1: DNA was extracted from amniotic fluid for the fetuses and from blood samples for the parents and children using QIAsymphony (Qiagen, Hilden, Germany) and fragmented using a L220plus (Covaris, PerkinElmer, Waltham, MA, USA). Libraries were prepared using the kits TruSeq DNA PCR-Free (Illumina), automaton MICROLAB Star (Hamilton, Reno, NV, USA), analysis TapeStation 4200 (Agilent, Santa Clara, CA, USA), quantification with Collibri Library Quantification Kit (Invitrogen, Waltham, MA, USA), or QuantStudio 5 real Time PCR Systems (Applied Biosystems, Waltham, MA, USA). Paired-end sequencing was performed on a NovaSeq 6000 (Illumina). After demultiplexing (bcl2fastq, Illumina), sequences were aligned on the human genome reference (GRCh38.p13 primary assembly, Gencode 32, GCA_000001305.2) with BWA-MEM (0.7.17). Single-nucleotide variants and indels were called with HaplotypeCaller de GATK (4.1.8.0). After the first filtering (artefacts filtered out), all the variants were annotated and then analyzed using Variant Effect Predictor (version 98.3) and the software CuteVariant v0.1.12.dev0. The in silico predictions used were based on the SIFT, PolyPhen2, and Mutation Taster algorithms. Frequency and occurrences in the controls were assessed on the ExAC, dbSNP, 1000 Genomes Project, ClinVar, HGMD, and in-house databases. The sequencing data were interpreted by GL and SC based on the ACMG recommendations.

### 2.2. Exome Sequencing

Family 2: DNA was extracted from amniotic fluid for the fetuses and from blood samples for the parents and child using QIAamp DNA minikit (Qiagen) and Qiasymphony automated blood kit (Qiagen), respectively. A massive parallel exome sequencing analysis was performed using Human Exome 2.0 comprehensive exome spike-incapture kit (Twist Bioscience, South San Francisco, CA, USA) with enrichment of coding exons and intron/exon junctions of known genes in the human genome. Illumina short-read sequencing of 150 bases was performed in paired ends on a NextSeq 2000 (Illumina). Quality data were generated from a local pipeline (Hg38). The minimal data quality guarantee was >99% at 10× coverage. SNVs and indels were identified and annotated on an outsourced pipeline (SeqOne, Hg19). A CNV analysis was performed using the SeqOne and local pipelines. Sequencing data were interpreted by JM and LR based on the ACMG recommendations.

Family 3: DNA was extracted from amniotic fluid for the fetuses and from blood samples for the parents. A massive parallel exome sequencing analysis was performed using the Twist Comprehensive Exome kit (Twist Bioscience) with enrichment of coding exons and intron/exon junctions of known genes in the human genome. Illumina short-read sequencing of 150 bases was performed in paired ends on NextSeq (Illumin). Quality data were generated from the GermlineFamily pipeline. The minimum data quality guarantee was >98% at 30× coverage. SNVs, indels, and CNVs were identified and annotated using the SeqOne pipeline. Sequencing data were interpreted based on the ACMG recommendations.

### 2.3. Clinical Data

Clinical data and medical history were retrospectively collected in local records.

### 2.4. Prenatal and Postnatal Imaging

All imaging data, prenatal and postnatal, ultrasound (US) and MRI, were initially interpreted or secondarily reviewed by SC and LG (radiologists).

### 2.5. Autopsy and Neuropathological Examination

According to French law, a fetal autopsy can be performed after the interruption of pregnancy if parental consent is obtained and based on standardized protocols. The examination includes X-rays, photographs, and macroscopic and histological examinations.

### 2.6. Immunohistochemistry

The fetus from Family 2 was tested for immunostaining on mesencephalic sections with antibodies against MPDZ to assess the proteic expression and location in the ependymal of the aqueduct of Sylvius. Immunostaining was performed using rabbit polyclonal antibodies against MPDZ with the automated slide stainer Benchmark GX (Ventana) according to the manufacturer’s instructions. The dilution was 1/800. Immunostaining was carried out at the same time in a control fetus without brain malformation, with normal histology of the midbrain paired for age.

## 3. Results

Th three families that were investigated for fetuses and children showing congenital ventriculomegaly were found to have genetic variants in their *MPDZ* genes (OMIM#603785). The imaging, genetics, and outcome data are summarized in Table 1.

### 3.1. Family 1

**Figure 1:**
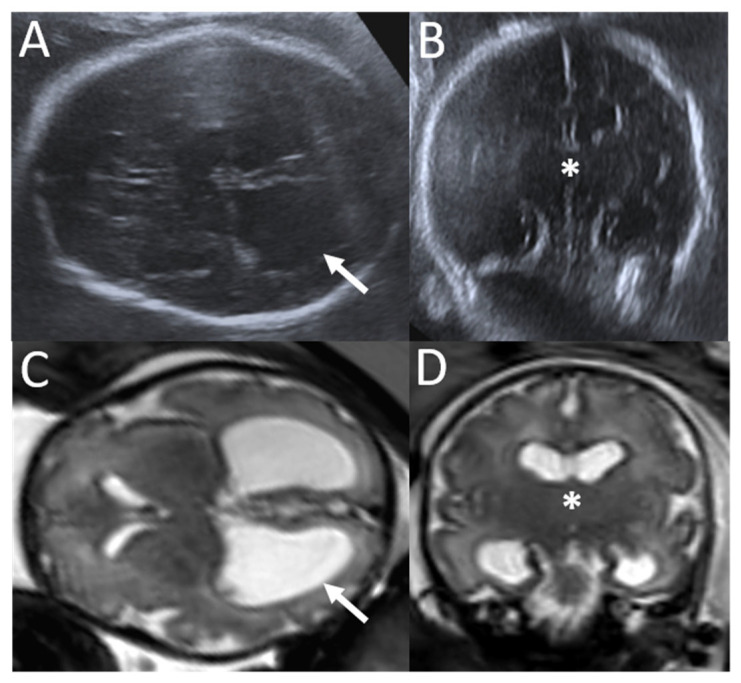
Fetal brain US ((**A**,**B**) using abdominal low-frequency probe) and MR ((**C**) axial, (**D**) coronal T2 weighted images) images at 33 WG showed dilation of the lateral ventricles predominantly posterior (arrows) without dilated third and fourth ventricles. Indeed, downstream from the ventriculomegaly, the third ventricle appeared atretic with an aspect of fused thalami on the midline (*).

**Figure 2:**
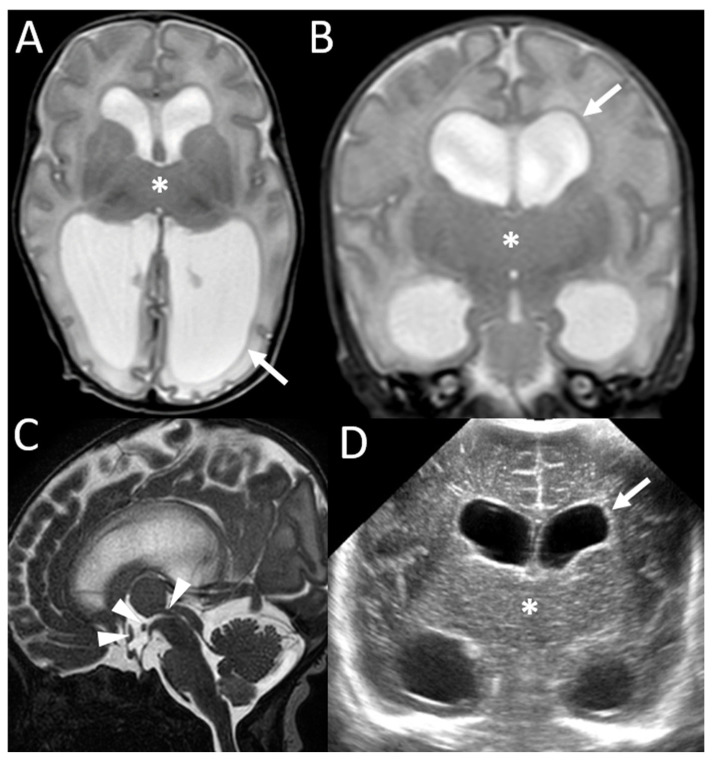
Postnatal MR image ((**A**) axial, (**B**) coronal, (**C**) sagittal T2 weighted images) at 21 days and transfontanellar US image at 1 day (**D**) confirmed progressive obstructive ventriculomegaly (arrows) upstream from an atretic third ventricle with an aspect of fusion of the thalami (*). Other parenchymal bridges were visible, crossing the midline in the third ventricle and the proximal aqueduct of Sylvius (arrowheads in C on mid-sagittal T2 drive images).

A 31-year-old pregnant woman, third gesture, was referred to our center at 33 weeks of gestation (WG). She had no personal medical history. The members of the couple are related (her mother was his second cousin). The female fetus had ventriculomegaly and intra-uterine growth retardation (IUGR) diagnosed at 22 WG. The atrium was measured at 11 mm for a normal range under 10 mm. Fetal weight was estimated under the 1st centile, and head circumference was measured at the 56th centile. A fetal neurosonogram and MRI confirmed mild ventriculomegaly involving both lateral ventricles, predominantly on their posterior part, and careful analysis of the diencephalon demonstrated partially fused thalami downstream from the ventriculomegaly (Figure 1). There was no evidence of intraventricular hemorrhage. Gyration was concordant with the gestational age. The midline and posterior fossa were otherwise normal. This female fetus was born at 34 WG in a context of IUGR. Head circumference at birth was 31.5 cm (−2 standard deviations (SD)) and increased after birth until +2 SD. A transfontanellar ultrasound confirmed progressive obstructive ventriculomegaly upstream from the partial fusion of the thalami and partial third-ventricle atresia (Figure 2D). MRI confirmed the sonographic features (Figure 2). No other CNS or extra-CNS anomaly was detected. The electroencephalogram was normal for age. Surgical ventriculocisternostomy was performed at 4 months of life, with a good surgical outcome. At the last examination, she was 20 months old and showed a mild delay in neurodevelopment, with progression of acquisitions (crawling, ten spoken words, and hypotonia). Head circumference was measured at +1 SD.

**Figure 3:**
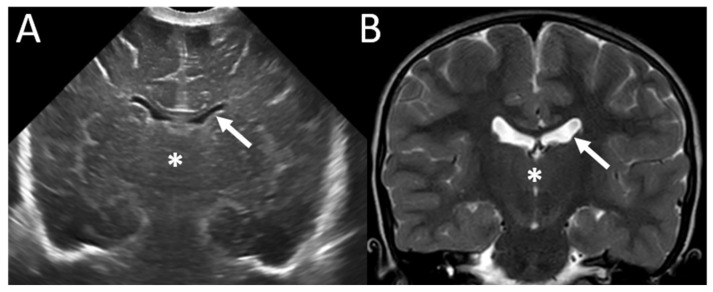
Postnatal transfontanellar ultrasounds (**A**) and brain MR ((**B**) coronal T2 weighted images) images, at 9 days and 4 years, respectively, showed the presence of an atretic third ventricle with aspects of partial fusion of the thalami on the midline (*) without ventriculomegaly (arrows).

The medical history of the second sibling included a mild isolated ventriculomegaly (5th month of pregnancy), which had improved postnatally, and led to a transfontanellar ultrasound being performed in the neonatal period. In keeping with the recent diagnosis of partial diencephalosynapsis for the last child, a retrospective review of the images (Figure 3A) showed also partial fusion of the thalami on the midline with partial third-ventricle atresia, which we confirmed by brain MRI, with reduced size of the third-ventricular lumen without any residual ventriculomegaly (Figure 3B). Head circumference was normal (+1 SD). At the last examination, at 4 years and 8 months old, neurodevelopment was normal (walking at 11 months, language between 1 and 2 years, and currently well-constructed sentences). He showed some mild attentional disorders at school. A psychometric assessment showed normal cognitive abilities (WISC-V).

A homozygous single-nucleotide variant was found in the *MPDZ* gene by genome sequencing in the two affected siblings, chr9(GRCh38)g.13136805T>C, NM_001378778.1:c.4201-2A>G, inherited from each parent (analysis in quatuor: both affected siblings and both parents). It affects the acceptor splice site, upstream from exon 30. In silico predictions predict exon skipping (SpliceAI: acceptor loss = 0.94). This variant was absent from the gnomAD_V4 population database. This variant was classified as pathogenic according to the ACMG recommendations.

### 3.2. Family 2

**Figure 4:**
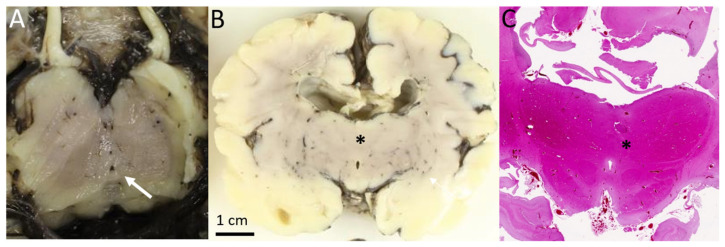
Fetopathology examination at the macroscopic (**A**,**B**) and microscopic (**C**) scales at 34 WG confirmed atresia of the third ventricle with a large fusion of the thalami (*) and superior atresia of the aqueduct of Sylvius (arrow).

A 27-year-old pregnant woman, fourth gesture, was referred at 28 WG for isolated fetal ventriculomegaly observed since 24 WG in a female fetus. The members of the couple are related. Fetal brain ventriculomegaly increased during pregnancy (measuring 13, 21, and 29 mm at, respectively, 24, 28, and 33 WG). Fetal brain MRI showed dilation of the lateral ventricles with a small third ventricle. The lumen of the aqueduct of Sylvius was not visible. Pericerebral fluid spaces were reduced, and the parenchyma was thin, with normal gyration, suggestive of an obstructive pattern. The absence of dilation of the third ventricle was associated with an aspect of partial fusion of the thalami on the midline. The fourth ventricle, vermis, and transverse cerebellar diameter were normal. There was no evidence of intraventricular hemorrhage. Due to initial prenatal counselling suggestive of a most likely poor neurological outcome, the couple opted for pregnancy interruption that was performed at 34 WG. The fetopathology examination confirmed predominantly posterior dilation of the lateral ventricles upstream from the large fusion of the thalami on the midline, with third ventricle atresia extending to the upper part of the aqueduct of Sylvius (Figure 4). Rosettes were observed in the aqueduct of Sylvius and the fourth ventricle. The cerebellum was normal apart from rare fusions of the foliations of the cerebellum. There was no extra-CNS anomaly.

Using an exome sequencing analysis, the fetus was found to be compound heterozygous for the non-sense variant chr9(GRCh38)g.13121778T>A, c.5191A>T, p.(Lys1731Ter) and the single-nucleotide variant chr9(GRCh38)g.13125214C>T, NM_001378778.1:c.4807+1G>A, affecting the donor splice site downstream from exon 35, in the *MPDZ* gene. Each variant was inherited from one parent. These variants were classified as pathogenic according to the ACMG recommendations. The c.4807+1G>A variant altered the consensus donor splice site and was predicted to lead to exon skipping (SpliceAI donor loss = 0.93).

**Figure 5:**
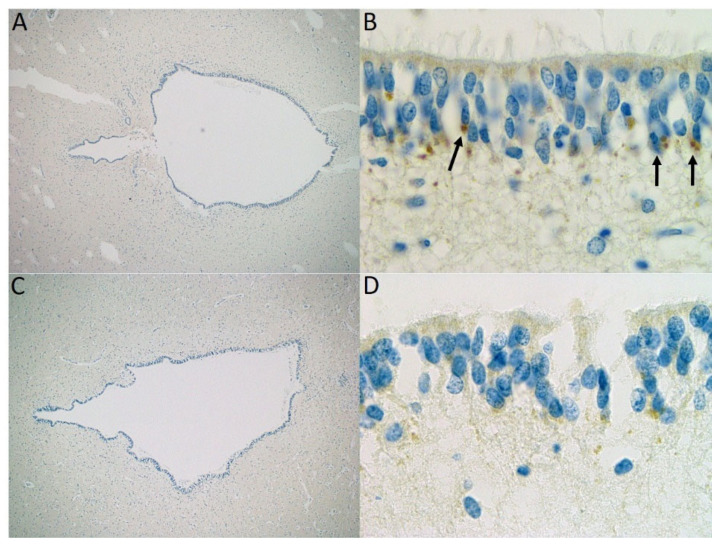
Immunostaining of the aqueduct of Sylvius using an MPDZ antibody in the fetus of Family 2 at 34 WG (**A**,**B**) and a control fetus of 35 WG (**C**,**D**) at magnification ×40 and ×1000: partial fusion of the walls of the aqueduct of Sylvius and formation of ependymal rosettes. Note the increased size and positivity of cytoplasmic granulations in the ependyma (arrows) related to the accumulation of abnormal MPDZ proteins in the patient.

The immunostaining with antibodies against MPDZ was positive in this fetus and showed an abnormal expression of MPDZ (Figure 5). In the control, cytoplasmic granulations of medium size were visible at the basal part of the ependymal under the nuclei and a more discreet staining at the apical cytoplasm. We observed increased size and intensity of basal granulations in the fetus carrying *MPDZ* variants compared to the control. Apical staining was considered the same in both the diseased and control fetuses. We interpreted this result as a maintained production of the MPDZ protein in the ependymal cells of the diseased fetus, but with accumulation of the abnormal protein either in the Golgi apparatus or in the endoplasmic reticulum, both located in the cytoplasm near the nucleus, often on the basal side.

The next pregnancy of the couple was marked by a recurrence of mild ventriculomegaly related to downstream partial diencephalosynapsis and third-ventricle atresia. The couple opted for pregnancy continuation. The newborn was born at 38 WG, vaginally, with a head circumference measured at 35 cm (0 SD), Apgar score 9/10/10/10. He shared the same *MPDZ* variants as the previous fetus. Seven days after birth, a congenital heart disease was diagnosed with multiple ventricular septal defects, and an hyperflow pulmonary arterial hypertension without abnormal pulmonary venous return, which required pulmonary cerclage. Postnatal brain transfontanellar US and MRI showed ventriculomegaly, fused thalami, and a few nodular unilateral periventricular neuronal heterotopias. At the last examination, the boy was 13 months old and showed a head circumference in the normal ranges as well as normal development (revised Brunet–Lézine scale, developmental quotient 94). Postnatal brain MRI confirmed mild and stable ventriculomegaly upstream from the partial third-ventricle atresia with aspects of partial fusion of the thalami. Systematic ophthalmological exploration showed bilateral macular dystrophy with no impact on visual behavior at 1 year old.

### 3.3. Family 3

A 35-year-old pregnant woman, fourth gestational period, was referred at 22 WG for isolated fetal ventriculomegaly in the context of a bichorial biamniotic pregnancy in two fetuses of different sexes. The members of the couple are not related. Fetal brain ventriculomegaly increased during pregnancy (measuring 13, 15, and 22 mm at 22, 24, and 26 WG for twin A and measuring 10, 18, and 22 mm at 22, 24, and 26 WG for twin B). The third and fourth ventricles were not dilated. Fetal brain MRI showed posteriorly predominant dilation of the lateral ventricles with a small third ventricle. The pericerebral fluid spaces were slightly reduced, and cortical gyration was normal, suggestive of an obstructive pattern. The absence of dilation of the third ventricle was associated with an aspect of partial fusion of the thalami on the midline in the two fetuses. The aqueduct of Sylvius was not visible. The fourth ventricle, vermis, and transverse cerebellar diameter were normal. There was no evidence of intraventricular hemorrhage. Due to initial prenatal counselling suggestive of a most likely poor neurological outcome, the couple opted for pregnancy termination that was performed at 28 WG. Fetopathology examination confirmed dilation of the lateral ventricles with septal rupture upstream from the third-ventricle atresia extending to the aqueduct of Sylvius. Rosettes were observed laterally to the aqueduct of Sylvius and to the fourth ventricle. The cerebellum was normal. There was no extra-CNS anomaly.

Using an exome sequencing analysis, the fetuses were found to be compound heterozygous for the intronic variants NM_001378778.1:c.5380-1G>C (intron 39) and NM_001378778.1:c.5725-1G>C (intron 43), affecting acceptor splice sites, in the *MPDZ* gene. Each variant was inherited from one parent. These variants were classified as probably pathogenic according to the ACMG recommendations. The variants were predicted for loss of the involved consensus acceptor splice sites.

## 4. Discussion

The MPDZ or multiple PDZ (PSD95, DLG, ZO-1) domain protein is a multidomain protein encoded by the *MPDZ* gene on 9p23, which is located in cellular tight junctions of epithelial cells, including those forming the choroid plexuses. It is also located on dendrites of neurons and is highly enriched in postsynaptic cells [4]. MPDZ domains are involved in multiple protein–protein interactions. *MPDZ* biallelic variant-induced ventriculomegaly is rarely reported in humans [5,6,7,8]. The underlying mechanism remains unclear, and several hypotheses have been brought forward to link *MPDZ* variants to the disease. Hydrocephalus related to *MPDZ* variants was initially considered non-progressive communicating hydrocephalus. The associated pathogenic hypothesis is based on the fact that MPDZ has been found to localize to tight junctions sealing the epithelial cells of the choroid plexuses. This presupposes that abnormal cell–cell adhesions are responsible for the uncontrolled secretion of cerebrospinal fluid within the ventricular system, leading to communicating ventriculomegaly [6]. However, this is not supported by our observations in which imaging features were highly suggestive of obstructive ventriculomegaly. The hypothesis would explain neither the absence of dilation of the third and fourth ventricles nor the obstructive pattern. In their *Mpdz*^−/−^ mice model, Feldner et al. showed morphologically normal tight junctions [9]. Moreover, the current understanding of CSF production by the choroid plexus is that it occurs through an active secretion process, which involves a complex interplay of inorganic ion exchange and relies on tight junctions to be efficient [10]. An alteration in the integrity of the epithelium would therefore lead to a reduction rather than an increase in CSF secretion. This is in line with the conclusion of Yang et al. who specified that no direct causative link had been established between possible MPDZ loss-of-function deleterious effects on intercellular junction integrity and the formation of hydrocephalus [11].

We evidence here an alternative hypothesis that comes from the interactions of the MPDZ protein with other molecular partners. MPDZ is functionally linked to the Crumbs polarity complex, including CRB2 (crumbs cell polarity complex component 2). Bi-allelic variations in *CRB2* were recently reported as a cause of obstructive congenital ventriculomegaly related to ventricular atresia, which could extend from the third ventricle to the aqueduct of Sylvius, the fourth ventricle, and, most caudally, up to the central canal of the medulla [12]. Ependymal anomalies, ependymal cells scattered in rosette-like structures of atresia-forking, and macrophagic reaction were described by Tessier et al. in this genetic form of ventriculomegaly with ventricular system atresia. This atresia was hypothesized to arise from an abnormal apical constriction of the ventricular cells of the neural tube corresponding to the future ependymal cells [12]. CRB2, MPDZ, and CCDC88C, another protein implicated in congenital ventriculomegalies, have been colocalized at the apical cell junction in the neural plate and cooperate to drive apical constriction of the neural plate cells during neurulation [12]. Therefore, they play an important role in the formation of the ventricular system and in the transformation of the primitive lumen into the central canal of the medulla. Immunostaining with antibodies against MPDZ in one fetus from our series confirmed a disorder in the ependymal cell location of MPDZ at the level of the aqueduct of Sylvius compared to the control. Considering the interaction between MPDZ and CRB2 and based on the above-mentioned pathogenic observations made in *CRB2* variants, we postulate that *MPDZ*-related ventriculomegaly is an obstructive form due to anatomical obstacles at the level of the third ventricle. This hypothesis is supported by our imaging analysis and by the data from the *Mpdz*^−/−^ mice model by Feldner et al., in which they demonstrated that the flow of cerebro-spinal fluid was blocked through the aqueduct of Sylvius [9]. Moreover, several authors observed multiple ependymal anomalies, some of which could be secondary to high intracranial pressure such as ependymal abrasions and astrogliosis [9,12], while others could correspond to the same mechanism of rosette formation as described in *CRB2* variants [8,12].

Both our imaging data and fetopathology examinations corroborate this hypothesis. Indeed, the description of our six patients and fetuses with *MPDZ* pathogenic variants, and the precise analysis of associated brain MRI data demonstrated an obstructive pattern of the ventriculomegaly upstream from the partial fusion of the thalami, also called diencephalosynapsis, with partial atresia of the third ventricle. A review of either published neuro-imaging figures or imaging reports of human and animal cases of hydrocephalus related to *MPDZ* led us to underline that fusion of the thalami and atresia of the third ventricle was frequently overlooked by authors. This reinforces the importance of a precise imaging analysis. Al-Dosari et al. noted that the third ventricle was not visualized in their case [6]. Shaheen et al. described an enlarged massa intermedia in one case [7]. Saugier-Veber et al. described absent or severely narrowed third ventricle with a collapse of the thalami [8]. The third ventricle was not dilated in *Mpdz*^−/−^ mice, with a median mass visible on the published images [11]. Thus, the identification of the often-overlooked fused thalami with partial third-ventricle atresia is a crucial anatomical clue to guide prenatal genetic testing when facing obstructive ventriculomegaly.

Such a fusion of the thalami leading to obstructive etiology should be attentively scrutinized by imaging when facing symmetrical dilation of the lateral ventricles associated with an obstructive pattern but without any dilation of the third ventricle. Such a pattern of ventricular dilation differs from the one of aqueduct obstruction characterized by a tri-ventricular dilation including the supra-pineal recess, in which thalami are pushed aside by third ventricle dilation upstream from an obstruction at the level of the aqueduct of Sylvius [13]. The latter pattern is characteristic of X-linked obstructive ventriculomegaly related to the pathogenic variant in the gene *L1CAM* (OMIM# 308840), even if rare cases of the *L1CAM* variant have been reported with diencephalosynapsis [14]. The literature on abnormal differentiation of the diencephalon leading to partial or complete fusion of the thalami and atresia of the third ventricle have remained scarce since its original publication under the term “diencephalosynapsis” on fetal imaging [15]. Pathological differentiation of the diencephalon, mesencephalon, and rhombencephalon during embryogenesis has been described as obstructive causes of early prenatal ventriculomegaly, which can be isolated (such as diencephalosynapsis with atretic third ventricle) or combined with mesencephalic obstruction at the level of the Sylvius aqueduct or with rhombencephalic anomalies such as rhombencephalosynapsis (fusion of the cerebellar hemispheres) [2]. De France et al. suggested that aqueduct anomalies could be either a malformative entity associated with diencephalosynapsis or secondary to the reduction of cerebro-spinal fluid flow downstream from the fused thalami [15]. Finally, the identification of diencephalosynapsis on prenatal imaging should prompt for screening for other malformations not only related to mesencephalic or rhombencephalic anomalies but also for anomalies that can orientate toward other genetic anomalies, such as corpus callosum agenesis or brainstem dysgenesis, characteristic of *RAC3* pathogenic variants [16].

Our data provide new information concerning the heterogeneity of clinical manifestation of the conditions induced by *MPDZ* variants, which may help in prenatal counselling. Regarding postnatal outcomes, our cases presenting with pathogenic variants of *MPDZ* (Family 1) showed an intrafamilial variability of both severity of the ventriculomegaly and outcome, ranging from mild delays to normal neurodevelopment. The severity of both ventriculomegaly and outcome was more variable in single cases from the literature (from very severe ventriculomegaly associated with stillbirths or death in early infancy to mild ventriculomegaly associated with normal neurodevelopment without surgical shunting) [5,6], and our series demonstrates that this heterogeneity is also observed despite the same genetic variations in siblings from the same family. Our data associated with those from the literature depict a broad phenotypic spectrum, with several patients showing grey matter heterotopias, with variable intra-familial penetrance [7], as shown in Family 2 of the present case series. Cases with seizures have been reported to be well-controlled under medication [17]. Some cases presented with facial dysmorphism, most likely related to the ventriculomegaly (frontal bossing). Ophthalmological anomalies have been reported in several patients such as chorioretinal or iris coloboma, foveal dysplasia, thin inner retina, and autosomal recessive maculopathies [6,7,17]. Some patients even presented with an eye-limited phenotype such as in one case from the family described by Iyengar et al., with an intra-familial ophthalmological phenotype variability [17,18]. Interestingly, MPDZ was found in the tight junctions of the retina, localized to the sub-apical region [17]. So, in fetuses with suspected MPDZ-related ventriculomegaly, chorioretinal colobomas should be attentively searched by imaging to orient the genetic diagnosis, particularly in the case of consanguinity. Likewise, an attentive and early ophthalmological examination should be proposed to patients with biallelic *MPDZ* variants to look for complications of intracranial high pressure in cases of severe ventriculomegaly but also for lesions related to the genetic anomaly.

Concerning the limitations of the present study, even if the small sample size suggests that *MPDZ*-related ventriculomegalies could be a rare condition, we are not able to provide an exact incidence of *MPDZ* variants among the multiple causes of congenital ventriculomegalies nor on potential minor forms that could lead to partial congenital diencephalosynapsis without upstream ventriculomegaly. The retrospective design, despite potential biases on the analysis and limitations on the nature of available biological samples, allowed us to gather several cases with a common molecular origin of ventriculomegaly and to find specificities on underlying anatomical mechanisms.

## 5. Conclusions

The analysis of imaging and fetopathological data of the six fetuses and children cases of congenital ventriculomegaly we report, related to *MDPZ* variants, led to clarify the underlying pathogenic mechanism. Ventriculomegaly related to *MPDZ* is a form of obstructive hydrocephalus, related to diencephalosynapsis and partial third-ventricle atresia, which can extend to the Sylvius aqueduct. This leads to reduced cerebro-spinal fluid flow at the level of the third ventricle with intra-familial variability of the impact on the size of lateral ventricles. On prenatal imaging, one should be aware of such etiology when facing an obstructive pattern with bilateral symmetrical dilation of the lateral ventricles without dilation of the third ventricle. A careful search for downstream partial or complete fusion of thalami with partial or complete third-ventricle atresia and for associated ocular anomalies is recommended.

## Figures and Tables

**Table 1:** Imaging, genetics, and outcome data.

	Pre and Post-Natal Imaging	Genetic Coordinates and Status for *MPDZ* Variant	Outcome
P1—Family 1	Mild ventriculomegaly (11 mm) Fused thalami IUGR	c.4201-2A>G, homozygous, inherited	Surgical ventriculocisternostomy at 4 months Mild delayed neudodevelopment at 20 months
P2—Family 1	Mild ventriculomegaly Fused thalami	c.4201-2A>G, homozygous, inherited	Post-natal spontaneous resolving of the ventriculomegaly Normal neurodevelopment at 4 years 8 months Mild attentional disorders at school Normal cognitive abilities
P3—Family 2	Severe ventriculomegaly (29 mm) Fused thalami Not visible aqueduct	c.5191A>T (p.(Lys1731Ter)), c.4807+1G>A, compound heterozygous, inherited	Teermination of pregnancy at 34 WG
P4—Family 2	Mild ventriculomegaly Fused thalami Few nodular unilateral periventricular neuronal heterotopias Congenital heart malformation	c.5191A>T (p.(Lys1731Ter)), c.4807+1G>A, compound heterozygous, inherited	Pulmonary cerclage Bilateral macular dystrophy Normal development at 13 months
P (twin 1)5—Family 3	Severe Ventriculomegaly (22 mm) Fused thalami Not visible aqueduct	c.5380-1G>C, c.5725-1G>C, compound heterozygous, inherited	TMedical termination of pregnancy at 28 (twin 2)WG
P6—FaSevere vily 3	Ventric(ulome)galy 22 mm Fused thalami Not visible aqueduct	c.5380-1G>C, c.5725-1G>C, compound heterozygous, inheTited	Medical termination of pregnancy at 28 WG

Abbreviations: IUGR: intra-uterine growth restriction; WG: weeks of gestation.

## Data Availability

Available on request.

## Abbreviations

CNS	central nervous system
IUGR	intra-uterine growth restriction
MRI	magnetic resonance imaging
SD	standard deviations
US	ultrasound
WG	weeks of gestation
